# Rosiglitazone infusion therapy following minimally invasive surgery for intracerebral hemorrhage evacuation decreases matrix metalloproteinase-9 and blood–brain barrier disruption in rabbits

**DOI:** 10.1186/s12883-015-0287-3

**Published:** 2015-03-17

**Authors:** Guofeng Wu, Junjie Wu, Yu Jiao, Likun Wang, Fan Wang, Yingjun Zhang

**Affiliations:** Emergency Department, Guizhou Medical University, No. 28, Guiyijie Road, Liuguangmen, Postal code 550004 Guiyang City, Guizhou Province People’s Republic of China; Department of Neurology of Affiliated Hospital, Guizhou Medical University, No. 28, Guiyijie Road, Liuguangmen, Postal code 550004 Guiyang City, Guizhou Province People’s Republic of China; Department of Medical Images of Affiliated Hospital, Guizhou Medical University, No. 28, Guiyijie Road, Liuguangmen, Postal code 550004 Guiyang City, Guizhou Province People’s Republic of China

**Keywords:** Intracerebral hemorrhage, Minimally invasive surgery, Rosiglitazone, PPARγ, MMP-9, Blood–brain barrier

## Abstract

**Background:**

The objective of this study was to investigate the effects of Rosiglitazone (RSG) infusion therapy following minimally invasive surgery (MIS) for intracerebral hemorrhage(ICH) evacuation on perihematomal secondary brain damage as assessed by MMP-9 levels, blood–brain barrier (BBB) permeability and neurological function.

**Methods:**

A total of 40 male rabbits (2.8–3.4 kg) was randomly assigned to a normal control group (NC group; 10 rabbits), a model control group (MC group; 10 rabbits), a minimally invasive treatment group (MIS group; 10 rabbits) or a combined MIS and RSG group (MIS + RSG group; 10 rabbits). ICH was induced in all the animals, except for the NC group. MIS was performed to evacuate ICH 6 hours after the successful preparation of the ICH model in the MIS and MIS + RSG groups. The animals in the MC group underwent the same procedures for ICH evacuation but without hematoma aspiration, and the NC group was subjected to sham surgical procedures. The neurological deficit scores (Purdy score) and ICH volumes were determined on days 1, 3 and 7. All of the animals were sacrificed on day 7, and the perihematomal brain tissue was removed to determine the levels of PPARγ, MMP-9, BBB permeability and brain water content (BWC).

**Results:**

The Purdy score, perihematomal PPARγ levels, BBB permeability, and BWC were all significantly increased in the MC group compared to the NC group. After performing the MIS for evacuating the ICH, the Purdy score and the ICH volume were decreased on days 1, 3 and 7 compared to the MC group. A remarkable decrease in perihematomal levels of PPARγ, MMP-9, BBB permeability and BWC were observed. The MIS + RSG group displayed a remarkable increase in PPARγ as well as significant decrease in MMP-9, BBB permeability and BWC compared with the MIS group.

**Conclusions:**

RSG infusion therapy following MIS for ICH treatment might be more efficacious for reducing the levels of MMP-9 and secondary brain damage than MIS therapy alone.

## Background

Primary intracerebral hemorrhage (ICH) accounts for 10 to 20% of stroke but has the highest rates of mortality and morbidity of all stroke subtypes [1]. Optimal management of spontaneous ICH remains one of the highly debated areas in the field of neurosurgery. Earlier studies comparing open surgical intervention with optimal medical management failed to show a clear benefit [2]. Recently reported evidence has shown that surgery is beneficial if it is performed early before the patient deteriorates [3]. Performing a surgical procedure 6–12 hours after ICH shows the most significant decrease in MMP-9, BBB permeability, and neurological deficit score [4]. However, the role of open surgical management of supratentorial ICH is still unresolved although decompressive craniectomy with hematoma evacuation might be a useful surgical procedure for selected patients with large hemispheric hypertensive ICH [5]. Minimally invasive techniques to evacuate clots appear to be a promising area of research and warrant further investigation [6]. Currently, minimally invasive surgery (MIS) is considered as a beneficial treatment for supratentorial spontaneous intracerebral hemorrhage [7]. MIS for ICH evacuation has been demonstrated to be an effective and safe method in clinical and experimental studies [8–16]. Patients with supratentorial intracerebral hemorrhage may benefit more from MIS than other treatment options [17]. However, MIS only reduces the brain damage caused by the hematoma to some extent and does not completely eliminate the damage [16]. The roles of MIS in reducing secondary brain damage remain limited as the erythrocytes and cytotoxic substances, which extravasate into the perihematomal brain, are difficult to completely remove. As a result, MIS to remove ICH followed by medications to prevent secondary brain damage might be another optimal choice [18, 19]. MIS plus a recombinant tissue-type plasminogen activator has achieved favorable results by reducing perihematomal edema [20].

Initial ICH is always followed by secondary brain damage, which is associated with a range of inflammatory factors, including matrix metalloproteinase-9 (MMP-9), a member of matrix metalloproteinase family (MMPs). MMPs have been demonstrated to play an important role in the disruption of the BBB after ICH [21,22]. MMP-9 participates in the dysregulation of BBB during hemorrhagic transformation, and it exacerbates brain injury after cerebral ischemia [23]. MMPs are critical for hematoma and brain edema growth as well as for neuronal death in hemorrhagic stroke [24]. MMP-9 may take part in the secondary injury after ICH, and it is positively correlated with perihematomal edema volume and the severity of ICH [25].

Perihematomal edema volume positively correlates with active MMP-9 and MMP-2 at 24 h and with active MMP-9 at 48 h [26]. The inhibition of MMP activity using pharmacological anti-MMP strategies and reducing the release of MMP-9 by MIS for ICH evacuation may provide an optimal approach for reducing ongoing edema after ICH [27].

RSG, an agonist of peroxisome proliferator-activated receptor-gamma (PPARγ), has been demonstrated to be effective in the treatment of ischemic stroke [28,29]. There are a few studies that have reported the role of RSG in the treatment of ICH [19,30], thus demonstrating that RSG promotes hematoma resolution, decreases neuronal damage, and improves functional recovery. These findings suggest that the resolution of hematomas induced by microglia/macrophages activated by PPARγ is a therapeutic target in ICH treatment [30]. In macrophages, PPARγ may act as an important factor in promoting hematoma absorption and protecting other brain cells from ICH-induced damage [19]. However, there is little information regarding the role of combining the MIS and RSG infusion therapy in the treatment of ICH. Studies have suggested that RSG intervention may downregulate MMP-9 expression by upregulating PPARγ expression. Activation of PPARγ in humans limits the expression of the matrix-degrading MMP-9 [31]. The administration of RSG following MIS to treat ICH might be more efficacious in decreasing the MMP-9 level to reduce secondary brain damage. Although RSG has been associated with an increased risk of stroke, the adjusted hazard ratio for RSG compared to pioglitazone is 1.27 for stroke [32]. However, the risk of hospital admission for stroke is not significantly affected by RSG use [33]. PPARγ agonists have been demonstrated to reduce recurrent stroke and total events of cardiovascular death or non-fatal stroke.

The present study was designed to assess the effect of RSG infusion therapy following MIS on perihematomal secondary brain damage in a rabbit model of ICH.

## Methods

### Main materials

#### Reagents

The following reagents were used in this study: pre-dyed protein marker (Fermentas, Ottawa, Canada), x-ray slice (Shanghai Kodak Company, Shanghai, China), SDS-PAGE gel kit (Beijing Kangwei Century Biological Technology, Beijing, China), TEMED (Sigma-Aldrich, California, USA), Tween 20, bovine serum albumin (BSA; Sigma-Aldrich, California, USA), 4 x protein sample buffer, beta-mercaptoethanol (Guiyang Saiweis Biotech Company, Guiyang City, China), PVDF membrane (Millipore, Massachusetts, USA), rabbit polyclonal antibody for PPARγ, rabbit polyclonal antibody for beta-actin (Wuhan Boster Company, Wuhan, China), and rabbit monoclonal antibody for MMP-9 (Abcam, Massachusetts, USA).

#### Main instruments

ZH-Lanxing B-Type rabbit stereotaxic apparatus (Huaibei Zhenghua Biological Instrument & Equipment,Xuzhou City, Jiangsu Province, China), CT provided by the Guizhou Medical University, G1315 A diode-array detector (DAD, Agilent Technologies, USA), gel-imaging system (Gene Genius), U-3010 UV–VIS spectrophotometer (Hitachi), liquid-removing device (ranges of 100–1000 ml, 20–200 ml, and 0.5-10 ml) (Canada BBI), and primer design software (Primer Premier 5.0), were used in this study.

### Experimental groups

The present study was approved by the Animal Care and Use Committee of Guizhou Medical University, and it was performed according to the criteria of good laboratory practice for drugs.

Forty male rabbits (2.8–3.4 kg) were provided by the Experimental Animal Center of Guizhou Medical University. The rabbits were randomly assigned to a normal control group (NC group; 10 rabbits), a model control group (MC group; 10 rabbits), a minimally invasive surgery group (MIS group; 10 rabbits) or a combined MIS and RSG group (MIS + RSG group; 10 rabbits).

### Animal preparations

#### Intracerebral hemorrhage model

**Figure 1:**
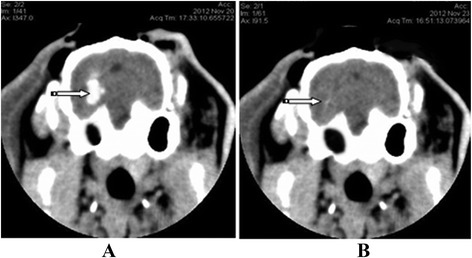
Brain CT showing ICH before and after minimally invasive surgery. **A**: The hyper-dense area (the arrow) in the brain CT showing successful preparation of the ICH model. **B**: The ICH was removed after minimally invasive surgery.

The method used to prepare an ICH model was the same as that used in our previously published studies [15,16,34]. Briefly, the rabbits were fastened to the stereotaxic apparatus, and their skulls were drilled. Using a #12 needle and a 1-ml syringe, 0.5 ml of autologous arterial blood was taken from the central ear artery. The syringe was then connected to a #7 needle from which the tip was removed. The #7 needle was then inserted vertically and quickly into the skull 12 mm deep, and the blood was slowly injected into the basal ganglia. A CT scan was performed 3 hours later. High-density regions in the basal ganglia were indicative of successful ICH induction (Figure 1A).

The rabbits were housed in the animal room after successful ICH induction as confirmed by CT scan. All of the animals recovered from anesthesia within 5 hours after intravenous injection of 20% urethane. The total anesthesia time was 3–5 hours.

The exclusion criteria included visualization of back flow along the needle track, blood in the ventricle, and death of the rabbit.

#### Minimally invasive surgery for intracerebral hemorrhage

The rabbits were anesthetized again by injecting 20% urethane (5 ml/kg) into the ear vein. The rabbits were then placed in the stereotaxic apparatus, and a #7 needle was inserted into the hematoma. The liquid part of the hematoma was aspirated, and 5,000 U of urokinase (dissolved in 0.5 ml of 0.9% sodium chloride solution) was injected into the hematoma. The needle was kept in place for 15 min followed by slow aspiration while withdrawing the needle. In the MIS + RSG group, RSG (0.5 mg dissolved in 0.5 ml of 0.9% sodium chloride solution) was infused into the hematoma regions after the ICH was removed. The procedures performed for the ICH evacuation were also conducted in the MC group but without aspirating the hematoma and without injecting urokinase into the hematoma. The rabbits were returned to the animal room after surgery.

#### Medical treatment of the animals

The animals in each group received an intramuscular injection of penicillin (400,000 U) to prevent infection, and they were housed in the animal until they were sacrificed. No other medical treatment was administered.

#### Evaluation of the intracerebral hemorrhage volume

A CT scan was performed on days 1, 3 and 7 to demonstrate the efficacy of the surgical procedures for ICH evacuation. The volume of the ICH was calculated before and after the MIS using the Tada formula as follows: π/6 × length (cm) × width (cm) × height (cm).

### Neurological deficit score measurement

A neurological deficit scale was used to compare the neurological function among groups [35]. The tests were conducted on days 1, 3 and 7 by an observer who was blinded to the treatments.

### Brain tissue preparation

All the animals were sacrificed on day 7 after the ICH model was successfully established. The brain was extracted and placed on ice. Using the needle track as the center to prepare a coronal section and a sagittal section, the brain was cut and divided into the following four parts on the hematoma side: anterior-inner, anterior-outside, posterior-inner and posterior-outside. From each part mentioned above, a total of 5 mm of brain tissue surrounding the hematoma was collected to determine the PPARγ level, MMP-9 level, EB content and brain water content (BWC).

### Peroxisome proliferator-activated receptor-gamma and matrix metalloproteinase-9

The brain samples were homogenized in a buffer containing Tris–HCl, NaCl, Triton X-100 and double-distilled water using a homogenizer. The mixture was centrifuged at 12000 rpm/min for 15 min at 4°C to isolate the supernatant. After protein quantification, the samples were separated by sodium dodecyl sulfate polyacrylamide gel electrophoresis (SDS-PAGE) and transferred onto a polyvinylidene difluoride (PVDF) membrane. The membrane was blocked with blocking buffer overnight at 4°C and incubated the next day with an anti-PPARγ (1:100) and anti-MMP-9 antibody (1:500) for one hour at room temperature followed by incubation with a secondary antibody (1:100). Finally, immunoreactive bands were visualized after incubation in a chemiluminescence substrate and exposure to film. β-actin (1:500) was used as an internal reference to normalize the results and was probed using the same procedures as PPARγ and MMP-9. The densitometric analysis of the bands was conducted using an image analysis system for Windows (National Institutes of Health, USA).

### Blood–brain barrier permeability measurement

Evans blue (EB) was used as a tracer to measure the BBB permeability. Two hours before each experiment, 2% EB (2 ml/kg) was injected into the ear vein. After 2 h, the brain tissue was quickly removed. The tissue surrounding the hematoma was weighed (with an accuracy of 0.1 mg) and then placed into a test tube with 4 ml of formamide.

The formamide method was used to measure the EB content in the brain tissue to gauge the severity of the BBB damage with the following formula: EB content in brain tissue (μg/g wet brain) = B × formamide (ml)/wet weight (g); where B is the EB content of the sample (μg/ml) given by the linear regression equation according to a standard curve.

### Brain water content measurement

The dry and wet weight method was used to measure the water content of the brain tissue. The brains were quickly removed, and a total of 5 mm of brain tissue surrounding the hematoma was collected. Brain tissues from the posterior-inner and posterior-outside parts of the hematoma were used for measuring the BWC. First, the weight of the wet tissue was obtained. The samples were placed in an oven at 100°C for 48 h, and the dried samples were then weighed. The water content of the brain tissue was calculated as follows: BWC = (wet weight – dry weight) / wet weight × 100%.

### Statistical analysis

All data were analyzed using SPSS 11.5. Basic data are expressed as the mean ± standard deviation (X ± SD). ANOVA was used to make comparisons among groups. A repeated measures ANOVA was used to make comparisons across the entire time series. When a difference was detected by ANOVA, the LSD test was used to make comparisons between two groups. A *p*-value less than 0.05 was considered to be statistically significant. Statistical analysis was performed in consultation with the Department of Biostatistics of Guizhou Medical University.

## Results

### Intracerebral hemorrhage model

Following the infusion of blood into the basal ganglia, the rabbits manifested with contralateral hemiplegia and were unable to walk or crawl. Additionally, the contralateral extremities were less responsive to noxious stimulation. The brain CTs showed an oval or round hyperdensity in the basal ganglia (Figure 1A), demonstrating that the ICH model in this study was successful and reliable. All the animals were kept alive until the experiment was terminated. Forty rabbits (each group included 10 rabbits) were used in the present study.

### Intracerebral hemorrhage volume

**Figure 2:**
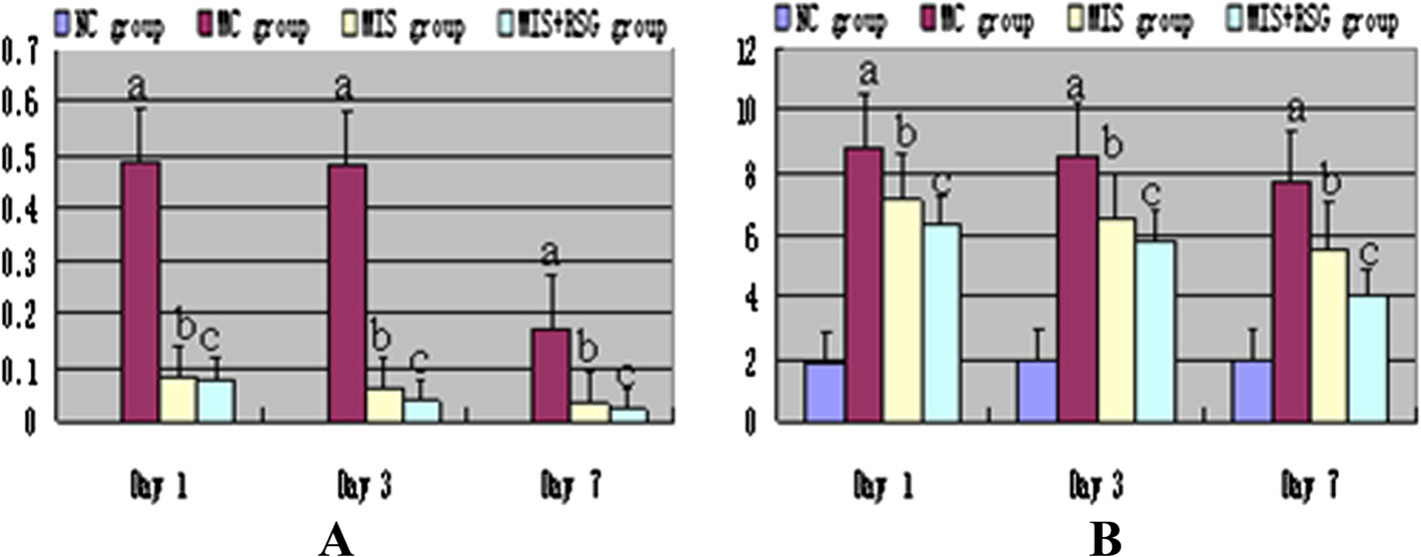
ICH volume and the Purdy score changes among different groups. **A**: The ICH volume changes after minimally invasive surgery (ml, $\overline{x}\pm s$). ^a^The ICH volume increased significantly compared to the NC group, *P* < 0.05; ^b^ Decreased significantly compared to the MC group, *P* < 0.05; ^c^ Decreased significantly compared to the MIS group or MC group, *P* < 0.05. **B**: The Purdy score changes after minimally invasive surgery (ml, $\overline{x}\pm s$). ^a^ The Purdy score increased significantly compared to the NC group, *P* < 0.05; ^b^ Decreased significantly compared to the MC group, *P* < 0.05; ^c^ Decreased significantly compared to the MIS group or MC group, *P* < 0.05.

There were no significant differences in hematoma volume among the MC (0.475 ± 0.022 ml), MIS (0.478 ± 0.025 ml) and MIS + RGS (0.480 ± 0.028 ml) groups after ICH was successfully induced. A significant decrease in the ICH volume was observed in the MIS and MIS + RSG groups at each time point (days 1, 3 and 7) compared to the MC group, thus suggesting that the MIS procedures successfully reduced intracerebral hematoma (Figures 1B and 2A). A significant difference was also observed in the ICH volume in the MC group among the three time points suggesting that the hematoma could be resolved and absorbed spontaneously.

### Functional neurological score

After the model of ICH was successfully established, the neurological deficit scores of the animals increased significantly (8.834 ± 0.753) compared to the normal control (2.000 ± 0.233) suggesting that the model produced a neurological deficit. In the MIS and MIS + RSG groups, the neurological deficit score decreased on days 3 and 7 compared to the MC group (Figure 2B). The MIS + RSG group displayed more favorable results compared with the MIS group.

### Peroxisome proliferator-activated receptor-gamma

**Figure 3:**
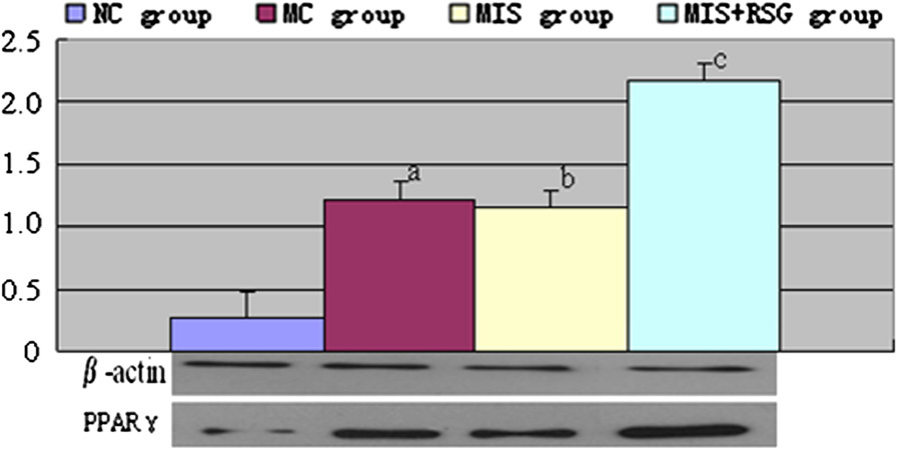
Changes of PPARγ protein expression among different groups (OD value, $\overline{x}\pm s$).^a^ The PPARγ protein increased significantly compared to the NC group, *P* < 0.05; ^b^ Decreased significantly in the MIS group compared to the MC group, *P* < 0.05; ^c^ Increased significantly compared to the MIS group or MC group, *P* < 0.05.

PPARγ was expressed in the normal rabbits, and the expression of PPARγ was significantly increased in the MC group suggesting that ICH might stimulate the microglia to increase PPARγ production. A significant difference was observed among the four groups (F = 108980.96 and *P* < 0.01). The LSD analysis demonstrated that the level of PPARγ protein decreased in the MIS group and increased in the MIS + RSG group compared to the MC group (*P* < 0.5). In the MIS group, the quantity of PPARγprotein was lower than the MIS + RSG group. These results suggested that the MIS decreased PPARγ protein expression after the ICH was reduced and that MIS followed by RSG treatment caused significant increases in the expression of PPARγ protein compared with the MC or the MIS group (Figure 3).

### Matrix metalloproteinase-9 level

**Figure 4:**
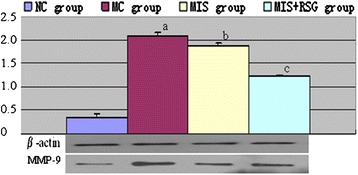
Changes of the MMP-9 among different groups (OD value, $\overline{x}\pm s$). ^a^ The MMP-9 increased significantly compared to the NC group, *P* < 0.05; ^b^ Decreased significantly in the MIS group compared to the MC group, *P* < 0.05; ^c^ Decreased significantly compared to the MIS group or MC group, *P* < 0.05.

A significant difference in perihematomal MMP-9 levels was observed among the NC, MC, MIS and MIS + RSG groups (F = 3236.992 and *P* < 0.001). MMP-9 was expressed at low levels in the NC group, and the expression of MMP-9 was significantly increased in the MC group. However, the level of perihematomal MMP-9 decreased significantly in the MIS and MIS + RSG groups compared with the MC group (Figure 4). The degree of decrease of perihematomal MMP-9 was more significant in the MIS + RSG group compared to the MIS group, thus suggesting that MIS followed RSG treatment was more efficacious in facilitating MMP-9 reduction.

### Evans blue and brain water content

**Figure 5:**
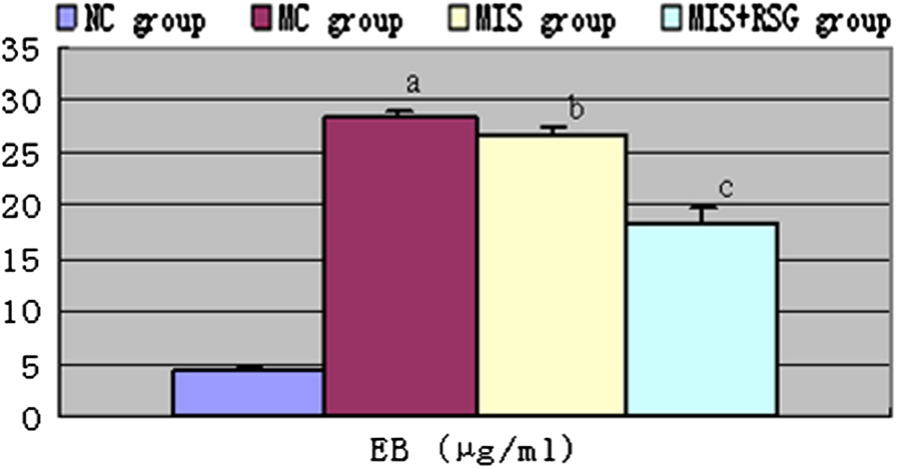
Changes of the Evans blue content among different groups (μg/ml, $\overline{x}\pm s$). ^a^ The Evans blue content increased significantly compared to the NC group, *P* < 0.05; ^b^ Decreased significantly in the MIS group compared to the MC group, *P* < 0.05; ^c^ Decreased significantly compared to the MIS group or MC group, *P* < 0.05.

**Figure 6:**
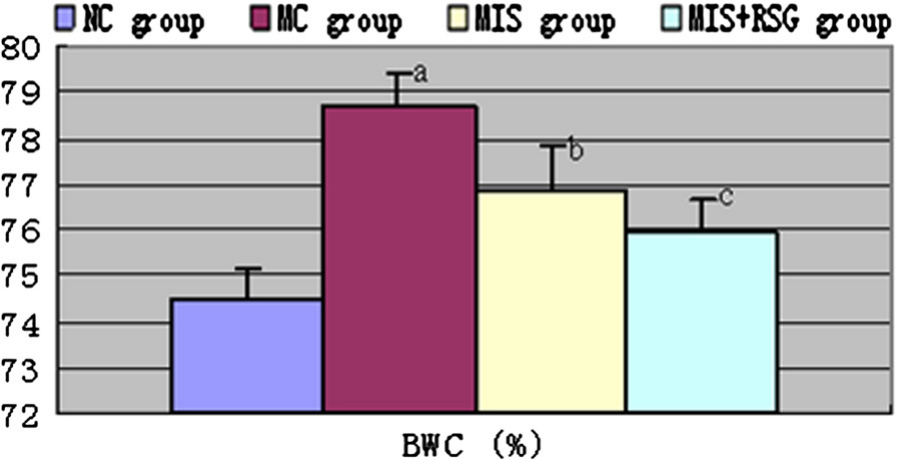
Changes of the BWC among different groups (%, $\overline{x}\pm s$). ^a^ The BWC increased significantly compared to the NC group, *P* < 0.05. ^b^ Decreased significantly in the MIS group compared to the MC group, *P* < 0.05; ^c^ Decreased significantly compared to the MIS group or MC group, *P* < 0.05.

Both the perihematomal EB (F = 495.501 and *P* < 0.001) and the BWC (F = 34.389 and *P* < 0.001) were significantly decreased in the MIS and MIS + RSG groups compared to the MC group. The MIS + RSG group displayed more significant decrease of EB content and the BWC compared to the MIS group. These results suggested that performing MIS procedures to evacuate the intracerebral hematoma decreases the EB and the BWC, and the results also showed that MIS followed by RSG treatment is more effective in reducing BBB permeability and cerebral edema (Figures 5-6).

## Discussion

In the present study, we utilized an animal model of ICH to investigate the effects of MIS followed by RSG treatment on perihematomal PPARγ levels, MMP-9 levels, EB content and BWC. These parameters were all significantly decreased in the MIS group compared to the MC group. These results suggested that the ICH-induced effects were obliterated after performing MIS and that the levels of neurotoxic substances, such as MMP-9, were reduced. As a result, the perihematomal inflammatory process was decreased, thus preventing the need of the microglia or the macrophages to produce more PPARγ to relieve the inflammatory damages after ICH. Accordingly, the levels of PPARγ and MMP-9 in the MIS group were significantly decreased compared to those of the MC group.

Compared to the MC and MIS groups, the PPARγ level was the highest in the MIS + RSG group, and the MMP-9 level, EB content and BWC were the lowest in the MIS + RSG group. These results demonstrated that the RSG infusion therapy increased the expression of PPARγ but decreased the MMP-9 level, EB content and BWC. RSG infusion therapy following MIS may be more efficacious in reducing MMP-9 levels and secondary brain damage than MIS therapy alone. Our previously published studies have demonstrated that performing MIS to evacuate ICH reduces the MMP-9 level and secondary brain damage [15,36]. Other investigators have demonstrated that PPARγ agonists may represent a potential strategy for the treatment of ICH [19]. In the present study, by combining MIS for ICH evacuation with PPARγ agonist infusion therapy directed at the ICH area, we achieved favorable results explained by more significant decrease of MMP-9 protein as well as the BBB permeability and the BWC, thus suggesting that this strategy would be more beneficial than the current method used for the clinical treatment of ICH.

In recent years, MIS has emerged as an alternative for traditional craniotomy due to its improved survival rate and reduced complication rate [37]. However, the utility of MIS for relieving secondary brain damage is limited as it cannot remove erythrocytes and cytotoxic substances, which extravasate into the perihematomal brain. Recent advances in understanding the pathogenesis of ICH-induced inflammatory injury have facilitated the identification of several novel therapeutic targets for the treatment of ICH. Potential therapies targeting secondary brain injury have increased interest in translational studies [38]. Although experimental studies have demonstrated that medications targeting secondary brain damage achieve favorable results, such strategy is unable to remove the occupying effect of the ICH. Consequently, performing MIS to remove the ICH followed by medications to prevent secondary brain damage may be another optimal choice [18,19].

MMP-9, one of the members of the MMP family, is associated with the disruption of the BBB and the formation of brain edema. Intraparenchymal thrombin induces brain edema formation through MMP-9 expression in rats. Accordingly, inhibiting the activity MMP-9 or decreasing its production may provide a potential approach for reducing ongoing edema after ICH [21]. Our previous studies have illustrated that MIS for ICH alleviation reduces the levels of MMP-9 to sufficiently reduce the permeability of the BBB but is unable to alleviate secondary brain damage [15,36]. Therefore, new effective therapies are urgently needed [39]. PPARγ agonists may be a potential strategy for the treatment of ICH. Intervention with RSG, a PPARγ agonist, may downregulate MMP-9 expression by upregulating PPARγ expression [40]. Thus, performing MIS for ICH evacuation followed by the infusion of PPARγ agonists into the hematoma region may be more effective in the treatment of ICH-induced secondary brain injury, which was demonstrated in the present study.

In this study, only one dose of RSG was tested. The effects of RSG at different doses and the side-effects on the brain were not investigated. Moreover, the sample size (10 rabbits/group) used in this study was also small. A further detailed experimental study is required to demonstrate the efficacy of PPARγ agonists following MIS for ICH treatment.

## Conclusions

The PPARγ level, MMP-9 level, BBB permeability and BWC increased after the ICH model was established, and these measurements were significantly decreased after the ICH was evacuated by MIS. Performing MIS followed by PPARγ agonist infusion therapy resulted in an increased PPARγ level and significantly decreased MMP-9 level, BBB permeability and BWC. Thus, the combination of the surgical removal of the hematoma with pharmacological treatment (via local infusion of RSG) to initiate the endogenous blood absorption mechanism to activate PPARγ is an optimal treatment strategy that is more efficacious for reducing secondary brain damage and improving neurological function than current treatment options.
